# The 24-Segment Global Sphericity Index Is Useful for Assessing Fetal Cardiac Function and as an Indicator of Fetal Well-Being

**DOI:** 10.7759/cureus.99253

**Published:** 2025-12-15

**Authors:** Hayato Kogita, Rie Oyama, Gen Haba, Hanae Kawamura, Tsukasa Baba

**Affiliations:** 1 Obstetrics and Gynecology, Iwate Medical University, Shiwa-gun, JPN

**Keywords:** fetal cardiac function, fetal heart quantification (hq), fetal well-being, global sphericity index, ultrasound

## Abstract

Background: Automated technologies have become increasingly vital in fetal cardiac assessment. Fetal heart quantification (HQ), which utilizes two-dimensional speckle tracking, provides quantitative measures of morphology and function. This study aims to identify the progression of many fetal cardiac function parameters during pregnancy in normal pregnant women and to determine which parameters are relevant to fetal well-being and worth considering.

Methods: We conducted a prospective observational study involving fetuses of 26 normal pregnant women to evaluate 27 fetal HQ parameters (including strain, sphericity indices, and four-chamber view measurements) across gestation. Factor analysis was used to identify indicators of fetal and neonatal well-being. Additionally, we analyzed a case of fetal growth restriction (FGR) complicated by a double-outlet right ventricle (DORV) and total anomalous pulmonary venous connection (TAPVC).

Results: Four parameters, which were tricuspid valve width, tricuspid valve length, the area of the four-chamber view, and right ventricular end-diastolic area (RVEDA), might be particularly informative. It is possible to perform serial assessments of fetal cardiac contractile development throughout gestation. In cases of FGR with DORV and TAPVC, fetal HQ detected abnormal right ventricular loading and impaired contractility. In cases of FGR with DORV and TAPVC, fetal HQ detected abnormal right ventricular loading and impaired contractility.

Conclusion: Fetal HQ provides a reproducible, quantitative approach to fetal cardiac assessment. These key indices may serve as potential indicators of fetal well-being and facilitate the early detection of cardiac dysfunction in high-risk pregnancies.

## Introduction

Fetal echocardiography is a vital tool for assessing fetal well-being and identifying congenital heart defects. However, the ability of conventional two-dimensional measurements of cardiac size and function in providing a comprehensive functional assessment is limited. To overcome these limitations, several novel approaches have been developed. For example, in 2016, DeVore et al. reported the differences between the point-to-point tracing method and the automated measurement method for evaluating the area and circumference of the fetal four-chamber view [1]. Subsequently, in 2018, the fetal four-chamber view was further subdivided into the apex, mid-heart, and base, with each region divided into eight segments: the apex (segments 1-8), mid-heart (segments 9-16), and base (segments 17-24). Based on this segmentation, the global longitudinal strain (GLS), calculated along the long axis of the fetal heart during diastole, has been shown to reflect ventricular contractile ability [2].

Furthermore, the measurement of 24-segment myocardial motion has been proposed as a novel index of cardiac function, and speckle-tracking echocardiography has enabled the quantitative evaluation of myocardial wall motion by analyzing the strain distance and angle between two points [3-5]. These approaches have expanded the possibility of assessing fetal cardiac function. Building on these advances, fetal heart quantification (HQ) has recently been introduced as a comprehensive ultrasound-based tool for the quantitative evaluation of the fetal heart [6,7]. Fetal HQ enables the simultaneous assessment of cardiac morphology, including size and shape, as well as the functional analysis of myocardial motion using strain and speckle tracking [4]. Compared to conventional methods, this approach provides a more detailed and reproducible evaluation of fetal cardiac function and has been increasingly applied in clinical studies. This study aims to identify the progression of fetal cardiac function in normal pregnant women (who are less affected by maternal cardiovascular conditions such as high blood pressure) and to determine which parameters are relevant to fetal well-being and are helpful.

## Materials and methods

**Figure 1:**
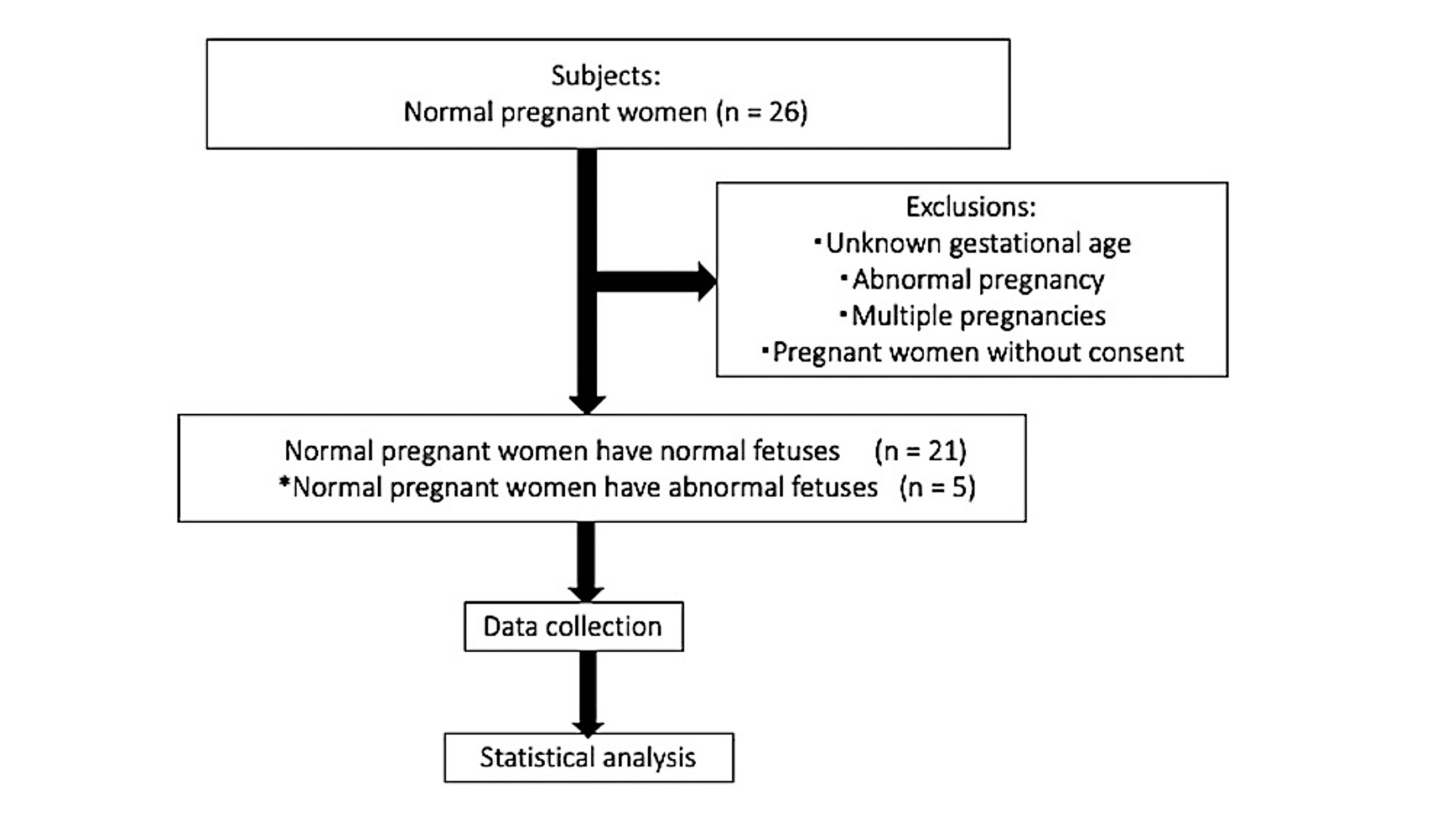
Flow from registration to analysis Twenty-six women with normal pregnancies were diagnosed at our hospital between October 2023 and July 2024. The inclusion criteria were singleton pregnancy without maternal complications, absence of fetal cardiac malformations, and no evidence of chromosomal abnormalities. The initial study subjects were women with uncomplicated pregnancies. *As an exception, they also included cases where fetal growth restriction or fetal cardiac anomalies were incidentally discovered during routine checkups; these cases were retained for continued examination using the fetal heart quantification software.

This is a longitudinal prospective cohort study of 26 fetuses with normal pregnancies diagnosed at Iwate Medical University Hospital in Shiwa-gun, Japan, between October 2023 and July 2024 (Figure 1). After obtaining informed consent, participants underwent ultrasound examinations at 18-39 weeks. If fetal cardiac abnormalities were suspected, they were referred to a pediatric cardiologist.

Inclusion criteria included singleton pregnancy without maternal complications, absence of fetal cardiac malformations, and no evidence of chromosomal abnormalities. Exclusion criteria comprised multiple pregnancies, incomplete clinical information, missed follow-up visits, and unknown gestational age. Despite these criteria, cases where fetal growth restriction (FGR) or fetal cardiac anomalies were incidentally discovered during routine checkups were retained and followed with ongoing ultrasound monitoring.

All examinations were performed using a GE Voluson Expert 22 ultrasound system (GE Healthcare, Zipf, Austria) equipped with a three-dimensional volumetric transducer (RM6C, 2-7 MHz) and integrated fetal HQ analysis software.

The apical four-chamber view was obtained with the apex directed toward the transducer whenever possible [7]. Instrument settings were optimized to enhance the delineation of the ventricular cavity and endocardial border. For each fetus, a cine loop of 2-3 s was recorded at a frame rate of ≥80 Hz.

Quantitative analysis was conducted using the fetal HQ software. Sampling points were manually placed at the junctions of the atrioventricular annulus and ventricular walls, as well as at the apex of the heart. The software automatically tracked the endocardial border throughout the cardiac cycle, enabling the assessment of myocardial strain and functional parameters.

The primary endpoints included several fetal cardiac function indices (Table 1). Secondary endpoints included gestational age at delivery, delivery method, birth weight, Apgar scores at one and five minutes, neonatal intensive care unit (NICU) admission, and neonatal death.

**Table 1:** Fetal cardiac function parameters Abbreviations and terms for fetal cardiac function parameters were listed on this table and article. Width 4CV and 4CV Trv. Width ED have the same meaning.

Category	Parameter	Segment/condition
Strain	LVGLS/RVGLS	Left/right ventricular global longitudinal strain
Fractional shortening	LVFS/RVFS	Left/right ventricle (global)
	LVFS1/LVFS12	Left ventricular segment 1/segment 12
	RVFS1/RVFS12	Right ventricular segment 1/segment 12
End-diastolic diameter	LVED	Left ventricle (global)
	LVED1/LVED12	Left ventricular segment 1/segment 12
	RVED	Right ventricle (global)
	RVED1/RVED12	Right ventricular segment 1/segment 12
End-district area	LVEA	Left ventricle (global)
	LVEA1/LVEA12	Left ventricular segment 1/segment 12
	RVEA	Right ventricle (global)
	RVEA1/RVEA12	Right ventricular segment 1/segment 12
End-systolic length	RVESL	Right ventricular end-systolic length
	LVESL	Left ventricular end-systolic length
Sphericity index	LVSI1/LVSI12	Left ventricular segment 1/segment 12
	RVSI1/RVSI12	Right ventricular segment 1/segment 12
Four-chamber view	Width 4CV or 4CV Trv. Width ED	Tricuspid valve width end-diastolic diameter
	Length 4CV	Four-chamber view length
	GSI 4CV	Four-chamber view global sphericity index
	Area 4CV	Four-chamber view area
Global function	EF	Ejection fraction
	EDV	End-diastolic volume
	ESV	End-systolic volume

All measurements were performed at least twice, and the mean values were used for analysis. Two senior physicians independently verified the image quality. Inter-examiner reliability was assessed using intraclass correlation coefficients (ICC). Data analyses were performed using IBM SPSS Statistics for Windows, Version 29.0 (IBM Corp., Armonk, New York, United States). Continuous variables were tested for normality and are expressed as medians (minimum-maximum) or as mean±standard deviation. Comparisons between normal and abnormal fetuses were performed using the Mann-Whitney U test. Regression analysis was conducted to evaluate the association between gestational age and cardiac parameters, with 95% confidence intervals (CI) presented in the regression figures. Factor analysis was also performed using IBM SPSS Statistics for Windows, Version 29.0. Statistical significance was set at p<0.05 or p<0.001.

This study was approved by the Ethics Committee of the Iwate Medical University Hospital (approval number: MH-2022-105). All procedures were conducted in accordance with the principles outlined in the Declaration of Helsinki.

## Results

The maternal characteristics are summarized in Table 2. In this study, 26 pregnant women underwent ultrasound examinations. The median maternal age was 33.23 years (range: 21-44), and the median BMI was 23.60 kg/m^2^ (range: 18.8-27.3). The median gestational age at ultrasound examination was 30.5 weeks (minimum-maximum: 18-39). Each participant underwent 1-7 ultrasound examinations during pregnancy, with a mean of 3.07 per participant, and for a total of 84 in all cases.

**Table 2:** Characteristics of the 26 uncomplicated pregnant women in the study Data are given as median (range) and median (minimum-maximum).

Maternal characteristics	
Number of cases	26
Age (years)	33.23 (range: 21-44)
BMI (kg/m^2^)	23.6 (range: 18.8-27.3)
Gravidity	1.0 (1.0-9.0)
Parity	0.0 (0.0-4.0)
Gestational age at ultrasound examination (weeks)	30.5 (18-39)
Total number of ultrasound examinations (times)	84
Median number of ultrasound examinations	3.0 (1.0-7.0)

In Table 3, we classified the fetuses into two groups: normal (n=21) and abnormal (n=5). Abnormal cases included congenital intrauterine infection (n=1), double-outlet right ventricle (DORV) (n=1), FGR with DORV with total anomalous pulmonary venous connection (TAPVC) (n=1), trisomy 21 (n=1), and intrauterine fetal death (n=1). In the normal group, delivery was by selective cesarean section (CS) in 12 cases (32.1%), emergency procedures in four cases (15.5%), and vaginal delivery in 10 cases (35.7%). In the abnormal group, CS was performed in one case (6%) and vaginal delivery in two cases (10.7%). Indications for CS in the normal group were selective (repeat CS: n=12) and emergency (non-reassuring fetal status: n=4). The abnormal group, however, showed only a single indication: emergency CS for non-reassuring fetal status (n=1). Gestational weeks and birth weight showed no significant differences between the normal and abnormal groups. The mean gestational weeks were 38.72±1.41 vs. 39.0±2.05 weeks (p=0.50), and the birth weights were 3041±13.16g vs. 2786.0±684.69 g (p=0.80). In contrast, Apgar scores at both one and five minutes were significantly different between the groups (p<0.01). Specifically, the one-minute scores were 7.86±0.40 vs. 6.60±0.61, and the five-minute scores were 8.87±0.89 vs. 8.46±1.9. Notably, the abnormal group included one case of intrauterine fetal death at 35 weeks of gestation.** **Abnormal cases required admission to the NICU; however, no neonatal deaths occurred.

**Table 3:** Characteristics of the 26 fetuses in the study Data are given as mean±standard deviation or percentage (%). *Mann-Whitney U test. (-) It is not applicable, because n=0.

Neonatal characteristics	Normal (n=21)	Abnormal (n=5)	P-value*
Gestational weeks at birth (weeks)	38.72±1.41	39.05±2.05	0.50
Vaginal delivery	10 (35.7%)	2 (10.7%)	0.02
Selective cesarean section	12 (32.1%)	0	-
Emergency cesarean section	4 (15.5%)	1 (6%)	0.30
Birth weight (g)	3054.0±313.16	2755.0±584.99	0.19
Apgar score at 1 minute	7.89±0.40	6.80±3.52	0.05
Apgar score at 5 minutes	8.97±0.60	8.45±1.90	0.05
Admission to the neonatal intensive care unit	0 (5)	4 (80%)	-
Neonatal death	0	0	-

Fetal cardiac parameters and gestational age

**Figure 2:**
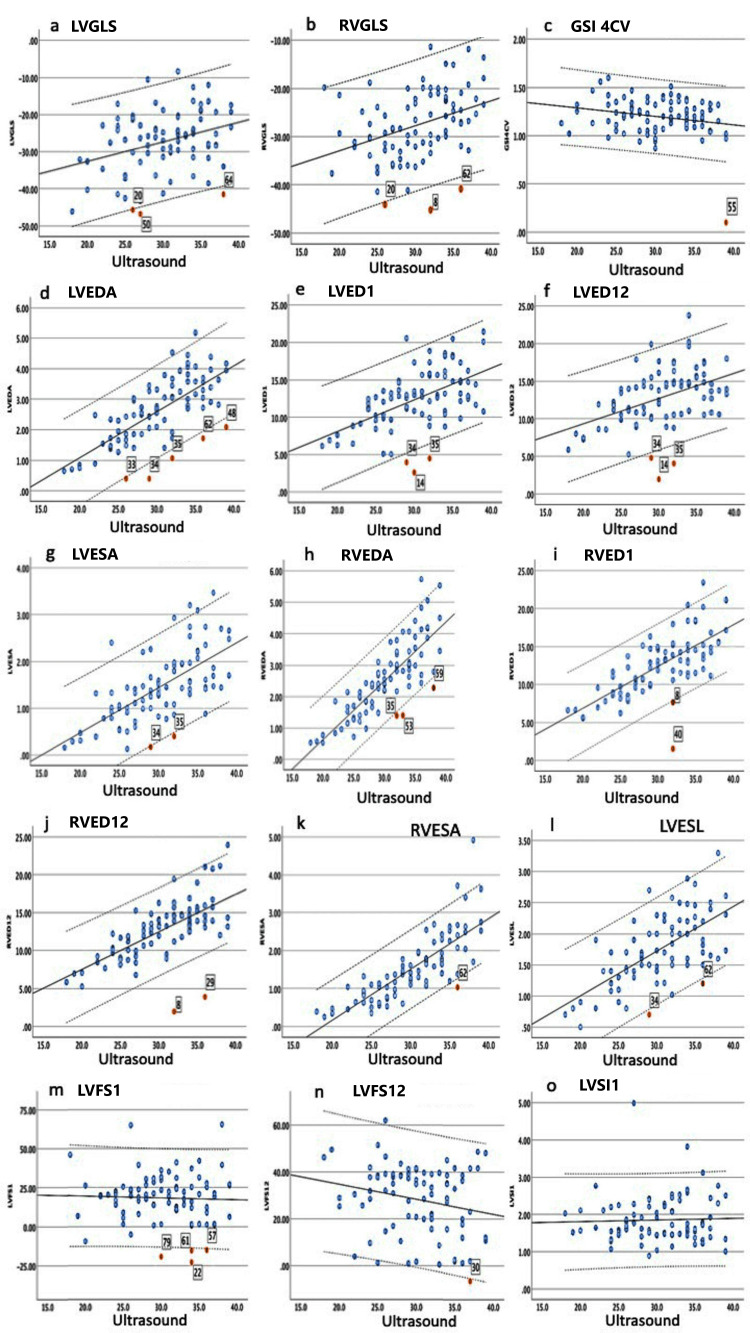
Regression line between 15 fetal cardiac function parameters and gestational age Linear regression for fetal cardiac parameters and gestational age with 95% CI in normal pregnancies. The number with a red circle represents cases outside the lower bound of the 95% CI (a-o). (a) LVGLS. (b) RVGLS. (c) GSI 4CV. (d) LVEDA. (e) LVED1. (f) LVED12. (g) LVESA. (h) RVEDA. (i) RVED1. (j) RVED12. (k) RVESA. (l) LVESL. (m) LVFS1. (n) LVFS12. (o) LVSI1. Ultrasound, which was an ultrasound examination at weeks of gestation. Unit of measurement: GLS (%), EDA and EDA (mm^2^), ESL and EDL (mm), and FS (%). Lines present a 95% CI. LVGLS: left ventricular global longitudinal strain; RVGLS: right ventricular global longitudinal strain; GSI 4CV: global sphericity index in the four-chamber view; LVEDA: left ventricular end-diastolic diameter area; LVED1: left ventricular end-diastolic diameter-segment 1; LVED12: left ventricular end-diastolic diameter-segment 12; LVESA: left ventricular end-systolic area; RVEDA: right ventricular end-diastolic area; RVED1: right ventricular end-diastolic diameter-segment 1; RVED12: right ventricular end-diastolic diameter-segment 12; RVESA: right ventricular end-systolic area; LVESL: left ventricular end-systolic length; LVFS1: left ventricular fractional shortening-segment 1; LVFS12: left ventricular fractional shortening-segment 12; LVSI1: left ventricular sphericity index-segment 1; GLS: global longitudinal strain; EDA: end-district area; ESL: end-systolic length; EDL: end-district length; FS: fractional shortening

**Figure 3:**
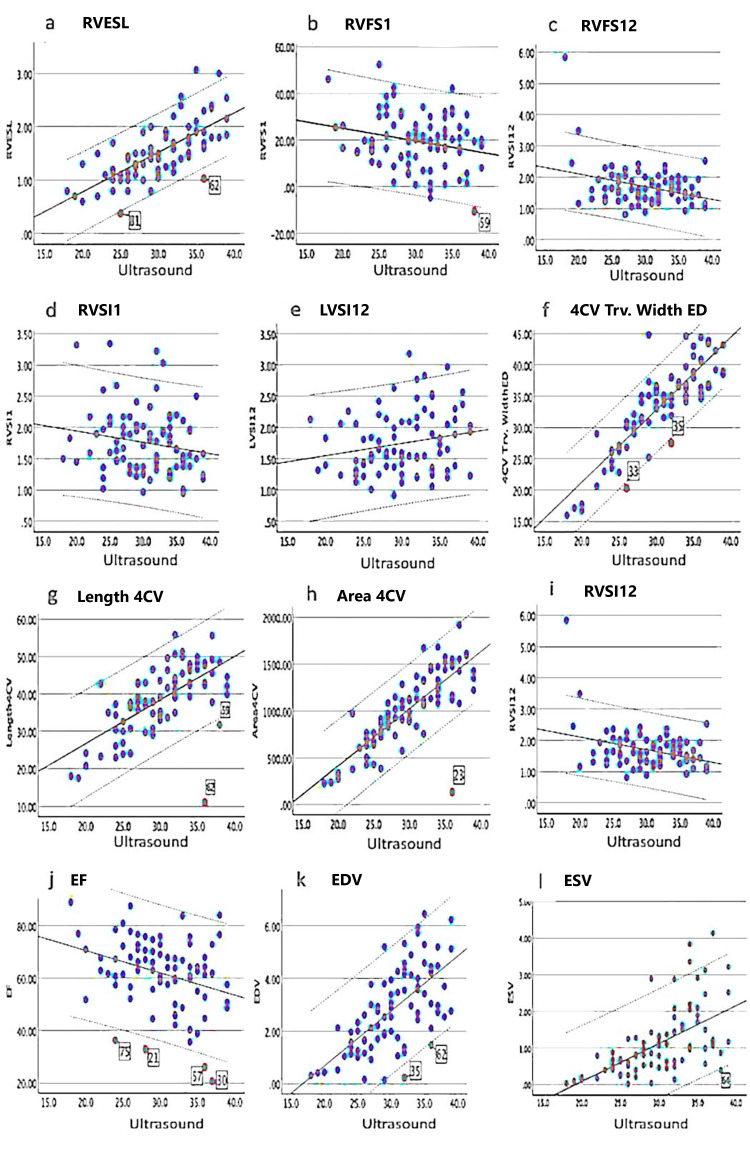
Regression line between 12 fetal cardiac function parameters and gestational age Linear regression for fetal cardiac parameters and gestational age with 95% CI in normal pregnancies. The number with a red circle represents cases outside the lower bound of the 95% CI (a-l). (a) RVESL. (b) RVFS1. (c) RVFS12. (d) RVSI1. (e) LVSI12. (f) 4CV Trv. Width ED. (g) Length 4CV. (h) Area 4CV. (i) RVSI12. (j) EF. (k) EDV. (l) ESV. Ultrasound, which was an ultrasound examination at weeks of gestation. Unit of measurement: area (mm^2^), length, ED and ESL (mm), FS and EF (%), and EDV and ESV (mL). Lines present a 95% CI. RVESL: right ventricular end-systolic length; RVFS1: right ventricular fractional shortening-segment 1; RVFS12: right ventricular fractional shortening-segment 12; RVSI1: right ventricular sphericity index-segment 1; LVSI12: left ventricular sphericity index-segment 12; 4CV Trv. Width ED: four-chamber view tricuspid valve width end-diastolic diameter; Length 4CV: four-chamber view length; Area 4CV: four-chamber view area; RVSI12: right ventricular sphericity index-segment 12; EF: ejection fraction; EDV: end-diastolic volume; ESV: end-systolic volume; ED: end-diastolic diameter; ESL: end-systolic length; FS: fractional shortening

We evaluated the relationship between gestational age and 27 fetal cardiac parameters in normal pregnancies using fetal HQ. Strong correlations were observed between gestational age and right ventricular end-diastolic diameter area (RVEDA) (r=0.82; p<0.001), right ventricular end-systolic area (RVESA) (r=0.80; p<0.001), and four-chamber view tricuspid valve width end-diastolic diameter (4CV Trv Width ED) (r=0.87; p<0.001). Moderate correlations were also found between gestational age and left ventricular end-diastolic diameter (LVED) (r=0.71; p<0.001), right ventricular end-systolic length (RVESL) (r=0.72; p<0.001), left ventricular end-systolic length (LVESL) (r=0.60; p<0.001), right ventricular end-diastolic diameter-segment 1 (RVED1) (r=0.71; p<0.001), right ventricular end-diastolic diameter-segment 12 (RVED12) (r=0.60; p<0.001), four-chamber view length (Length 4CV) (r=0.65; p<0.001), four-chamber view area (Area 4CV) (r=0.79; p<0.001), end-diastolic volume (EDV) (r=0.66; p<0.001), and end-systolic volume (ESV) (r=0.58; p<0.001). A weak correlation was observed for left ventricular end-diastolic diameter-segment 1 (LVED1) (r=0.54; p=0.046). On the other hand, the global sphericity index in the four-chamber view (GSI 4CV) (r=0.23; p=0.38), left ventricular global longitudinal strain (LVGLS) (r=0.32; p=0.003), and right ventricular global longitudinal strain (RVGLS) (r=0.36; p=0.001) were not significantly correlated with gestational progression (Figure 2a-2o, Figure 3a-3l, and Table 4). In Figures 2-3, the number with a red circle represents cases outside the lower bound of the 95% CI. 

**Table 4:** Evaluation of gestation age and cardiac functional parameters The values represent the 95% CI. *Regression analysis x: independent variable LVGLS: left ventricular global longitudinal strain; LVED: left ventricular end-diastolic diameter; LVESA: left ventricular end-systolic area; LVESL: left ventricular end-systolic length; RVGLS: right ventricular global longitudinal strain; RVEDA: right ventricular end-diastolic area; RVESA: right ventricular end-systolic area; RVESL: right ventricular end-systolic length; LVFS1: left ventricular fractional shortening-segment 1; LVFS12: left ventricular fractional shortening-segment 12; LVED1: left ventricular end-diastolic diameter-segment 1; LVED12: left ventricular end-diastolic diameter-segment 12; LVFS1: left ventricular fractional shortening-segment 1; LVSI12: left ventricular sphericity index-segment 12; RVFS1: right ventricular fractional shortening-segment 1; RVFS12: right ventricular fractional shortening- segment 12; RVED1: right ventricular end-diastolic diameter-segment 1; RVED12: right ventricular end-diastolic diameter-segment 12; RVSI1: right ventricular sphericity index-segment 1; RVSI12: right ventricular sphericity index-segment 12; 4CV Trv. Width ED: four-chamber view tricuspid valve width end-diastolic diameter; Length 4CV: four-chamber view length; GSI 4CV: four-chamber view global sphericity index; Area 4CV: four-chamber view area; EF: ejection fraction; EDV: end-diastolic volume; ESV: end-systolic volume

Parameters	Regression*	r*	95%CI			P-value*
				Lower	Upper	
LVGLS	y=21.46+3.53x	0.32	0.064	0.068	0.321	0.003
LVED	y=35.57+0.19x	0.71	0.367	2.621	4.079	<0.001
LVESA	y=23.82+4.40x	0.65	0.567	3.277	5.532	<0.001
LVESL	y=20.09+5.84x	0.65	0.758	4.339	7.356	<0.001
RVGLS	y=37.25+0.25x	0.36	0.072	0.110	0.396	<0.001
RVEDA	y=21.22+3.54x	0.82	0.277	2.995	4.099	<0.001
RVESA	y=22.97+4.74x	0.80	0.397	3.952	5.530	<0.001
RVESL	y=19.50+6.97x	0.72	0.744	5.498	8.459	<0.001
LVFS1	y=30.50-0.02x	0.04	0.036	-0.084	0.059	0.728
LVFSI12	y=32.38-0.07x	0.22	0.037	-0.148	-0.001	0.046
LVED1	y=21.63+0.69x	0.54	0.119	0.457	0.930	<0.001
LVED12	y=22.62+0.59x	0.45	0.132	0.336	0.861	<0.001
LVFSI1	y=29.73+0.29x	0.04	0.900	-1.501	2.081	0.748
LVSI12	y=26.61+2.08x	0.20	1.111	-0.122	4.298	0.064
RVFSI1	y=32.20-0.09x	0.23	0.046	-0.190	-0.007	0.035
RVFS12	y=32.11-0.09x	0.23	0.046	-0.189	-0.007	0.035
RVED1	y=18.89+0.90x	0.71	0.708	0.710	1.108	<0.001
RVED12	y=19.33+0.86x	0.66	0.111	0.649	1.089	<0.001
RVSI1	y=33.30-0.74x	0.18	1.063	-3.839	0.391	0.109
RVSI12	y=34.66-2.59x	0.33	0.8434	-4.253	-0.935	0.003
4CV Trv. Width ED	y=8.78+0.65x	0.87	0.041	0.567	0.731	<0.001
Length4CV	y=16.37+0.35x	0.65	0.047	0.266	0.452	<0.001
GSI4CV	y=37.29-5.86x	0.23	2.774	-11.378	-0.340	0.038
Area4CV	y=19.60+0.01x	0.79	0.001	0.009	0.012	<0.001
EF	y=35.58-0.12x	0.32	0.039	-0.196	-0.041	0.003
EDV	y=24.39+2.08x	0.66	0.262	1.561	2.603	<0.001
ESV	y=26.56+3.23x	0.58	0.503	2.227	4.230	<0.001

Cardiac parameters of cases in the low side of the 95% CI and background

Cardiac function parameters were below the lower limit of the 95% CI in 15 fetuses. In particular, in cases 1-4, many cardiac function parameters repeatedly fell below the lower limit of the 95% CI throughout pregnancy. However, cases 1 and 2 did not have significant fetal cardiac abnormalities. Case 3 was diagnosed with a chromosomal abnormality (trisomy 21) after birth. Case 4 was diagnosed with intrauterine growth restriction and congenital heart malformation and was admitted to the NICU after birth. Cases 1-3 were not admitted to the NICU. Even in the normal group (cases 5-15), the small-number parameters showed values below the lower limit of the 95% CI during pregnancy. These parameters included LVGLS, RVGLS, GSI 4CV, LVED1 and RVED1, left ventricular end-diastolic diameter-segment 12 (LVED12), right ventricular end-diastolic diameter-segment 12 (RVED12), left ventricular fractional shortening-segment 1 (LVFS1), left ventricular fractional shortening-segment 12 (LVFS12), left ventricular end-diastolic area (LVEDA), and ejection fraction (EF). Cases 11 and 13 showed low GLS or EF values between 24 and 30 gestational weeks before delivery, and fetal heart rate decelerations were recorded on cardiotocography during labor (Table 5).

**Table 5:** Cardiac parameter of cases in the lower confidence limit of 95% CI and patient background LBW: low birth weight; w: gestational weeks; CS: cesarian section; RFS: reassuring fetal status; DORV: double-outlet right ventricle; TAPVC: total anomalous pulmonary venous connection; FGR: fetal growth restriction; IUFD: intrauterine fetal death; NICU: neonatal intensive care unit; Apgar score: point at one minute/point at five minutes. (-): not admitted to the NICU RVGLS: right ventricular global longitudinal strain; LVGLS: left ventricular global longitudinal strain; Width 4CV: four-chamber view tricuspid valve width end-diastolic diameter; EF: ejection fraction; LVFS12: left ventricular fractional shortening-segment 12; Area 4CV: four-chamber view area; LVFS1: left ventricular fractional shortening-segment 1; LVFS12: left ventricular fractional shortening-segment 12; GSI 4CV: four-chamber view global sphericity index; Length 4CV: four-chamber view length; RVFS1: right ventricular fractional shortening-segment 1; RVEDA: right ventricular end-diastolic area; RVED1: right ventricular end-diastolic diameter-segment 1; RVED12: right ventricular end-diastolic diameter-segment 12; EDV: end-diastolic volume; RVESA: right ventricular end-systolic area; LVEDA: left ventricular end-diastolic area; LVGLS: left ventricular global longitudinal strain; ESV: end-systolic volume; LVESL: left ventricular end-systolic length; LVED1: left ventricular end-diastolic diameter-segment 1; LVED12: left ventricular end-diastolic diameter-segment 12; LVESA: left ventricular end-systolic area; RVFS12: right ventricular fractional shortening-segment 12

Case	Maternal characteristics	Delivery and infant characteristics	Cardiac parameters outside 95% CI at gestational age (weeks)	Apgar score	Umbilical cord blood pH	Admission to NICU	Remarks or indication of CS
1	33-year-old, primigravida	Selective cesarean at 38 weeks, male 3050 g	26w: RVGLS. 28w: RVGLS, Width 4CV, EF. 34w: LVSI12, Area 4CV, LVFS1. 36w: Area 4CV	8/10	7.27	-	Repeat CS, umbilical cord entanglement
2	30-year-old, multipara	Vaginal delivery at 39 weeks, female 3016 g	36w: Width 4CV, EF, LVFS1, LVSI12. 38w: GSI4CV, Length 4CV, RVFS1, RVEDA	8/9	7.36	-	Variable deceleration, RFS
3	44-year-old, multipara	Vaginal delivery at 40 weeks, female 3216 g (trisomy 21)	34w: LVFS1, LVSI12. 36w: RVGLS, Length 4CV, EDV, RVES, LVEDA, RVESA. 38w: LVGLS, ESV	8/9	7.36	-	Trisomy 21
4	35-year-old, multipara	Emergency cesarean at 40 weeks; male 780 g (LBW with DORV and TAPVC)	26w: LVEDA. 29w: LVESL, LVEDA, LVED1, LVED12, LVESA. 32w: LVSI12, LVEDA, LVED1, LVED12, LVESA, RVEDA, RVFS12, EDV	4/8	7.16	Admission	FGR, RFS
5	38-year-old, multipara	Selective cesarean at 37 weeks, female 2890 g	32w: RVGLS, RVED1, RVED12	8/10	-	-	Repeat CS, giving birth at another facility
6	34-year-old, multipara	Selective cesarean at 37 weeks, male 3226 g	30w: LVED1, LVED12	8/9	7.27	-	Repeat CS, healthy
7	35-year-old, primigravida	Emergency cesarean at 40 weeks, female 4094 g	36w: RVED12	8/9	7.35	-	RFS, healthy
8	35-year-old, primigravida	Emergency cesarean at 40 weeks, female 3514 g	37w: LVFS12, EF	8/9	7.16	-	RFS, healthy
9	43-year-old, primigravida	Emergency cesarean at 41 weeks, female 3294 g	32w: RVED1	8/9	7.31	-	RFS, healthy
10	31-year-old, multipara	Vaginal delivery at 39 weeks, female 2734 g	39w: LVEDA	8/9	7.31	Admission	Intrauterine infection, healthy
11	23-year-old, primigravida	Vaginal delivery at 39 weeks, female 3030 g	27w: LVGLS	8/9	7.26	-	RFS, healthy
12	35-year-old, multipara	Vaginal delivery at 39 weeks, female 3432 g	39w: GSI 4CV	8/9	-	-	Healthy
13	28-year-old, primigravida	Vaginal delivery at 38 weeks, female 3122 g	24w: EF	6/6	7.27	Admission	Prolonged deceleration, healthy
14	26-year-old, primigravida	Vaginal delivery at 35 weeks, male 2158 g	30w: LVFS1	-	-	-	IUFD
15	35-year-old, multipara	Vaginal delivery at 40 weeks, male 3016 g	25w: RVESL	8/9	7.27	Admission	Prolonged deceleration

Factor analysis

**Figure 4:**
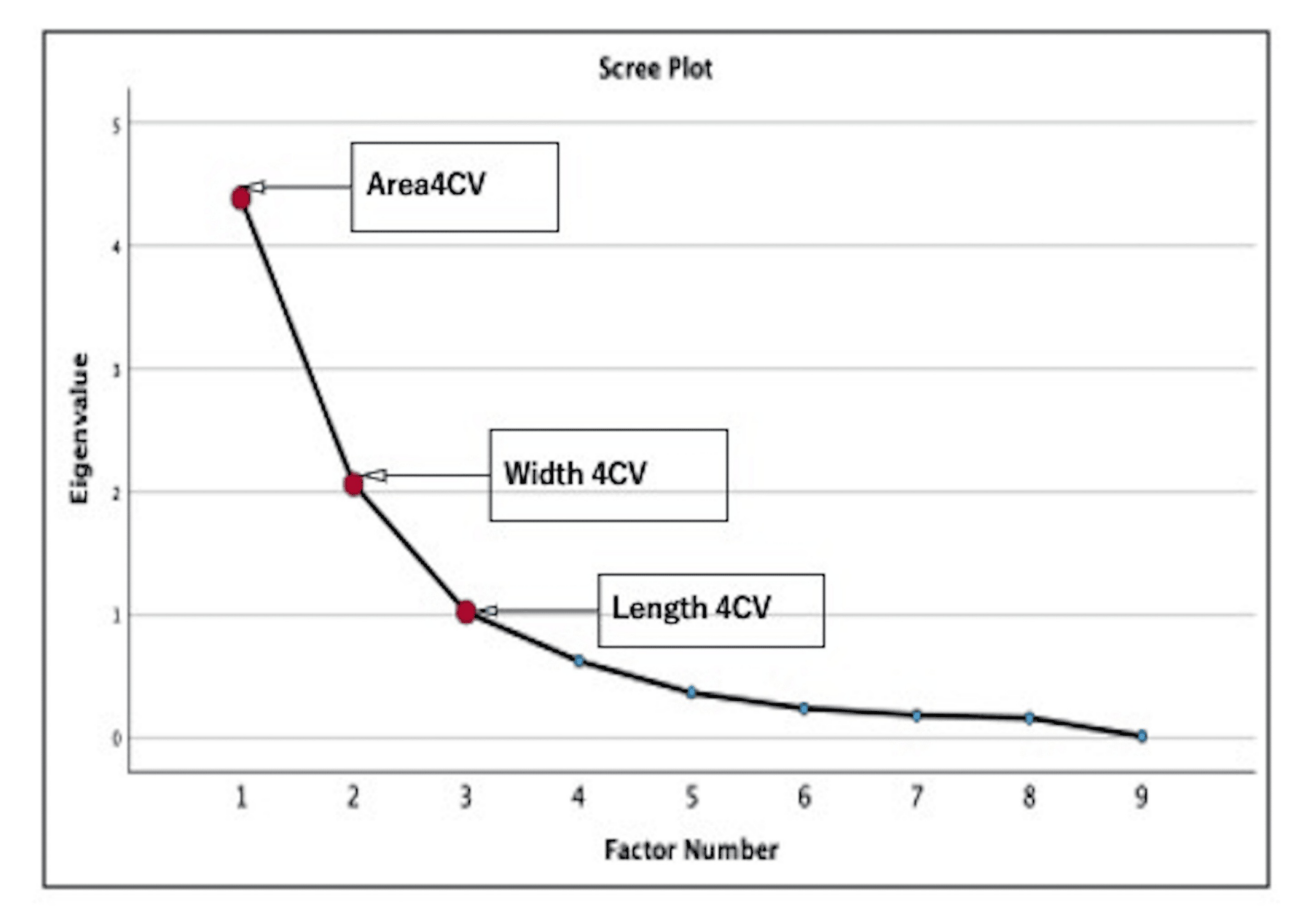
Scree plot obtained from factor analysis Three factors were determined: Area 4CV, Width 4CV, and Length 4CV. Area 4CV: four-chamber view area; Width 4CV: four-chamber view tricuspid valve width end-diastolic diameter; Length 4CV: four-chamber view length

Factor analysis was conducted to evaluate the validity of the cardiac function parameter using analysis software (IBM SPSS Statistics for Windows, Version 29.0), which was supported by the Kaiser-Meyer-Olkin (KMO) measure of sampling adequacy and Bartlett's test, which revealed that four-chamber view area, four-chamber view tricuspid valve width, end-diastolic diameter, and four-chamber view length were valid factors that were independent and valid indicators for assessing fetal cardiac function in normal pregnancies (Tables 6-7 and Figure 4).

**Table 6:** Factor matrix Extraction of fetal cardiac function parameter validity. The method used was principal axis factoring. Attempted to extract three factors. More than 25 iterations required (convergence=0.010). Area 4CV: four-chamber view area; Width 4CV: four-chamber view tricuspid valve width end-diastolic diameter; Length 4CV: four-chamber view length; LVESL: left ventricular end-systolic length; EF: ejection fraction; RVFS1: right ventricular fractional shortening-segment 1; RVGLS: right ventricular global longitudinal strain; GSI 4CV: four-chamber view global sphericity index

	Factor		
	1	2	3
Area 4CV	0.929	0.174	0.118
Width 4CV	0.896	0.138	0.016
Length 4CV	0.799	0.099	0.141
LVESL	0.795	-0.010	0.107
EF	-0.370	0.046	0.297
RVFS1	-0.227	0.664	-0.183
RVFS12	-0.283	0.549	0.065
RVGLS	0.354	-0.105	-0.632
GSI 4CV	-0.036	-0.102	0.296

**Table 7:** KMO measure of sampling adequacy and Bartlett's test KMO measure of sampling adequacy and Bartlett's test, which revealed that Area 4CV, GSI 4CV, and length 4CV were valid factors. Sig. indicates p-value, which is 0, indicating that the p-value was less than 0.001. KMO: Kaiser-Meyer-Olkin; Area 4CV: four-chamber view area; Width ED: four-chamber view tricuspid valve width end-diastolic diameter; GSI 4CV: four-chamber view global sphericity index; Length 4CV: four-chamber view length

KMO measure of sampling adequacy		0.805
Bartlett's test of sphericity	Approx. chi-squared value	346.394
	df	36
	Sig.	0

Reliability analysis

Interobserver reliability was assessed using the ICC. The single-measurement ICC was 0.760, and the average measurement ICC was 0.961 (p=0.001), indicating reproducibility (Table 8).

**Table 8:** Intra-examiner test Sig indicates p-value. The p-value of the intra-examiner test in this study was 0.001.

	Intraclass correlation	95% CI		F test with true value 0			
		Lower bound	Upper bound	Value	df1	df2	Sig
Single measures	0.760	0.22	1	26.283	1	7	0.001
Average measures	0.962	0.693	1	26.283	1	7	0.001

## Discussion

In normal pregnancies, the end-diastolic diameter (ED), end-systolic area (ESA), and end-systolic length (ESL) of both fetal ventricles, as well as the tricuspid valve width end-diastolic diameter (4CV Trv. Width ED), length (Length 4CV), and area (Area 4CV) measured in the four-chamber view (4CV), were positively correlated with advancing gestational age; furthermore, 4CV Trv. Width ED, Length 4CV, and Area 4CV could be proposed as valid parameters for assessing fetal well-being.

Fetal HQ

Fetal HQ, an automated technology that enables the quantitative assessment of fetal heart morphology and function, is based on two-dimensional speckle tracking, which allows real-time endocardial tracking independent of angle [8]. This technique is used to evaluate the size, shape, and function of the fetal heart by dividing it into 24 segments, enabling the quantitative analysis of cardiac function [1]. Traditionally, fetal heart size has been assessed using chamber dimensions or cardiothoracic ratios, primarily derived from the four-chamber view [3]. The American Heart Association recommends measuring the ventricular basal-to-apical diameter at end-diastole for assessing ventricular size [9]. In the present study, we analyzed 27 fetal cardiac function parameters obtained using fetal HQ across gestational ages in 26 normal pregnancies. We investigated their value as indicators of fetal and neonatal well-being. Factor analysis identified three parameters: 4CV Trv. Width ED, Length 4CV, and Area 4CV. We also found strong correlations between RVEDA, RVESA, and 4CV Trv. Width ED, as well as advanced gestational age in normal pregnancies. These findings are broadly consistent with the physiology of fetal circulation.

Fetal circulation

The present results are consistent with the basic description of the fetal circulation by Rudolph [10], Rudolph and Heymann [11], and Inamura [12]. In late-gestation fetal sheep, biventricular output is approximately 450 mL/kg/min, of which 200 mL/kg/min is delivered to the umbilical-placental circulation and 250 mL/kg/min to the fetus [13]. The right ventricle contributes approximately two-thirds of the total stroke volume, while the left ventricle contributes the remainder. Thus, during fetal life, the right ventricle exhibits significant circulatory function. The unique characteristics of fetal myocardium help explain the results of this study. Fetal myocardium contains a high proportion of noncontractile proteins (approximately 60% compared to approximately 30% in adults), fewer sarcomeres and T-tubules, and irregular fiber orientation [14]. This results in weak contractile force, limited preload reserve, and a rapid plateau in contractile force with increasing afterload [12]. This suggests that the right ventricular myocardial fibers are more longitudinally aligned, supporting the phenomenon of longitudinal shortening [4]. In other words, the fiber orientation of the left and right ventricles is collectively reflected in the circumferential orientation of the 4CV, which is composed of the longitudinal and transverse directions. 

FGR and congenital heart defects

Although our primary focus was on normal pregnancies, we included a case of FGR complicated by DORV and TAPVC. FGR, the failure of a fetus to reach its genetic growth potential because of maternal, placental, or fetal factors, is a significant cause of perinatal morbidity and mortality and is associated with long-term cardiovascular risk [15-18]. DORV, a complex CHD in which both great arteries arise predominantly from the right ventricle, accounts for 1-1.5% of all reported congenital heart disease and occurs in approximately one per 10,000 live births [19]. TAPVC, in which all pulmonary veins drain abnormally into the systemic veins or the right atrium, accounts for <1% of CHD and can be classified into supracardinal, cardiac, intracardiac, and mixed subtypes [20,21]. In our case of FGR with DORV and TAPVC, the fetal circulatory volume increased with gestation and growth, imposing a progressive load on the right ventricle. This phenomenon is associated with decreased RVEDA and right ventricular end-diastolic volume (RVEDV), which may reflect a decrease in right ventricular contractility that cannot accommodate the preload caused by altered shunting via the ductus arteriosus and intraventricular blood volume due to the septal defect. These findings suggest that fetal HQ parameters help detect pathophysiological changes in normal pregnancy and in complex congenital heart disease associated with FGR.

Limitations and future perspectives

This study has some limitations, including a small sample size and a single-center design, which may limit generalizability. In addition, only one case involved FGR with congenital heart disease, making it challenging to assess pathological conditions. Future multicenter studies with larger cohorts are needed to validate our findings and to clarify the role of fetal HQ parameters in high-risk pregnancies.

## Conclusions

Fetal HQ offers a highly reproducible, quantitative, and visual means of assessing fetal cardiac function. The right ventricle, which serves as the primary driver of fetal circulation, and four-chamber measurements (including tricuspid valve width, end-diastolic diameter, length, and area) change with gestation. These parameters reflect fetal well-being and cardiac physiology, potentially enabling the early detection of cardiac dysfunction in conditions such as preeclampsia and FGR. Despite its small sample size, this study is crucial for identifying which fetal HQ parameters are valid and relevant to fetal well-being in normal pregnant women (those unaffected by hypertension or other circulatory conditions). We anticipate that larger data analyses will further validate these initial findings and advance the field.
